# Emergence of CD26+ Cancer Stem Cells with Metastatic Properties in Colorectal Carcinogenesis

**DOI:** 10.3390/ijms18061106

**Published:** 2017-05-23

**Authors:** Alvin Ho-Kwan Cheung, Deepak Narayanan Iyer, Colin Siu-Chi Lam, Lui Ng, Sunny K. M. Wong, Hung-Sing Lee, Timothy Wan, Johnny Man, Ariel K. M. Chow, Ronnie T. Poon, Roberta Pang, Wai-Lun Law

**Affiliations:** 1 Department of Surgery, Li Ka Shing Faculty of Medicine, University of Hong Kong and Queen Mary Hospital, Sassoon Road, Pokfulam, Hong Kong; cheung_hokwan@hotmail.com (A.H.-K.C.); u3003219@connect.hku.hk (D.N.I.); siuchi88@gmail.com (C.S.-C.L.); lui.ng.612@gmail.com (L.N.); h0994148@hku.hk (S.K.M.W.); hungsinghk@yahoo.com.hk (H.-S.L.); u3002375@hku.hk (T.W.); johnnyb@hku.hk (J.M.); ariel115@gmail.com (A.K.M.C.); poontp@hku.hk (R.T.P.); 2 Centre for Cancer Research, University of Hong Kong, Pokfulam, Hong Kong

**Keywords:** colorectal cancer, metastasis, cancer stem cells, CD26

## Abstract

Colorectal cancer results from genetic aberrations which accumulate over a long period of time, with malignant and metastatic properties acquired at a relatively late stage. A subpopulation of CD26+ colorectal cancer stem cells are known to be implicated in metastasis. We quantified CD26+ cancer cells in 11 primary tumor samples by flow cytometry, and showed that tumors having confirmed or suspected metastases harbored a relatively high CD26+ level in these samples. We hypothesized that this subpopulation of cancer stem cells arises in the late stage of carcinogenesis from the bulk of tumor daughter cells which are CD26−. The manipulation of *PIK3CA* and *TP53*, two genes commonly deregulated in the late stage, had an effect on the maintenance of the CD26+ cell population. When CD26− tumor daughter cells were sorted and cultured, the emergence of tumor spheres containing CD26+ cells occurred. These findings shed light to the origin of colorectal cancer stem cells with metastatic properties, which has an implication on conventional treatments by surgery or adjuvant chemotherapy for tumor debulking.

## 1. Introduction

Patients with colorectal cancer may suffer from grave prognostic implications when metastatic disease develops. The understanding of cancer metastasis has evolved with the cancer stem cell model, which proposes that cancer is maintained and initiated by a rare population of tumor cells having unique self-renewal properties [1]. Recently, our group has discovered that a subpopulation of CD26+ cancer cells has stem-like features and may play a crucial role in leading to liver metastasis in colon cancer [2]. These cells are found preferentially in clinically metastatic liver tissues, having an increased ability to generate metastasis in mouse models accompanied with a higher resistance to traditional chemotherapy [2,3]. Importantly, a high percentage of CD26+ tumor cells in colorectal cancer was shown to correlate with a higher tumor stage and poor survival [4]. 

The prevailing model of colorectal cancer suggests that carcinogenic and metastatic properties are acquired in a stepwise process, with accumulations of new genetic aberrations [5]. Given that CD26+ colorectal cancer stem cells (CSC) with metastatic properties are preferentially found in metastatic tissues and advanced colorectal cancers with poor prognosis [2], we hypothesized that CD26+ CSC are generated during the later stage of carcinogenesis, where the cancer cells acquire a new genetic aberration leading to a CD26+ phenotype. 

We focused on genes which likely play a role in the later stage of carcinogenesis, such as *TP53* and *PIK3CA*. Mutations in both these genes are considered late events and occur in a substantial proportion of colorectal cancer, with approximately 70% of sporadic tumors harboring *TP53* mutations [6] and around 25% harboring *PIK3CA* mutations [7,8].

The current study focused on the possible origin and mechanism of maintenance of CD26+ colorectal cancer cells, which were shown to be CSC with metastatic properties. We quantified CD26+ in tumor samples by flow cytometry and correlated the findings with clinical data, in order to investigate if a high CD26+ level correlates with a later stage of cancer. We evaluated the functional roles of *PIK3CA* and *TP53* on CD26+ tumor cell maintenance. We hypothesized the possibility that these CSC with metastatic properties may arise from non-stem tumor cells. Finally, we discussed how our findings may apply to the current models of colorectal carcinogenesis.

## 2. Results

### 2.1. Primary Tumors with Confirmed or Suspected Metastases Harbor a Relatively High Proportion of CD26+ Cancer Stem Cell Subpopulation

**Figure 1:**
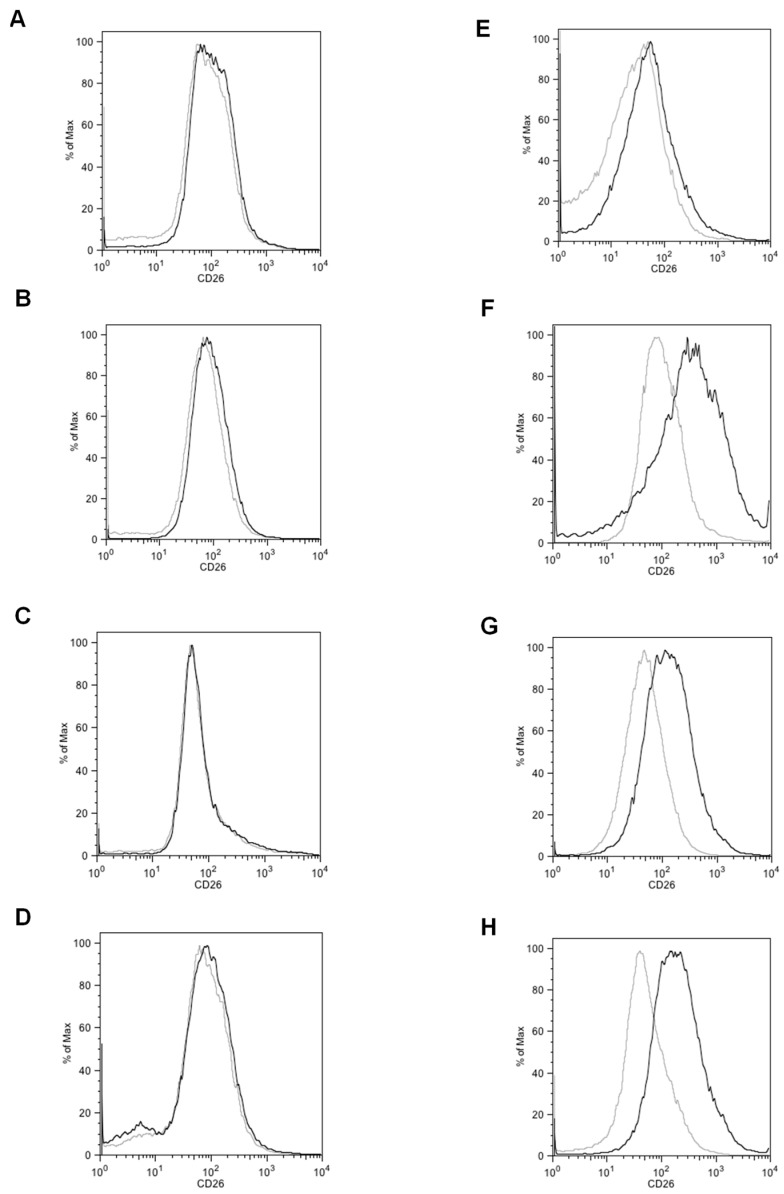
Representative flow cytometry plot of tumour sample of patients with (**A**–**D**, patients 2–5) lower than median, (**E**, patient 6) median, and (**F**–**H**, patients 9–11) higher than median level of CD26+ cancer cells. Grey line, isotype control. Black line, samples stained with CD26 antibody. The percentage of CD26+ cancer cells in each patient (defined by >99th percentile of CD26 intensity stained with isotype control) is listed in Table 1.

To investigate the notion that CD26+ cells appear in the later stage of colorectal carcinogenesis, we identified the percentage of CD26+ cells within clinical specimens from various tumor stages by flow cytometry. Our group has previously demonstrated that the number of primary tumors with liver metastases harboring >1% of CD26+ CSC was greater than that of primary tumors without metastases [2]. We have also showed that a higher proportion of CD26+ on immunohistochemistry correlates with a higher tumor stage and a poorer survival rate [4]. In this study, we performed flow cytometry on clinical specimens to quantify the accurate percentage of CD26+ cancer cells within the samples and subsequently correlated this result with the patient clinical data (Figure 1 and Table 1).

Clinical information and the percentage of CD26+ subpopulation in 11 patients are shown in Table 1. At the time of surgery, one patient had stage I disease, four had stage II disease, two had stage III disease, and four were known to have metastases (stage IV). These four patients all underwent resection of both the primary colorectal tumor and the liver (patients 7, 8, 9) or lung metastases (patient 6). For patient 9, new lung metastases were detected 19 months after the operation. For all patients, the percentage of CD26+ cancer cells in the primary colorectal resection specimen was 5.35 ± 5.38% (range = 0.2–13.2%). Tumors of stages I to III did not appear to show a trend of increasing CD26+ populations with stage in the analyzed cohort. Meanwhile, when the six patients with tumors harboring >1% CD26+ cell population were examined (patients 6–11), four of them were already diagnosed with metastatic disease (stage IV) at the time of surgery as mentioned above; the other two patients who had a particularly high proportion of CD26+ tumor cells had an initial pathological stage of IIA according to the TNM classification. However, patient 11 (Table 1), whose tumor consisted of 13.2% CD26+ cells, was later found to have liver metastases on ultrasonography 5 months after tumor resection and subsequently died. Meanwhile, patient 10 (Table 1), whose tumor harbored 12.7% CD26+ population, was found to have a suspected lung metastasis on reassessment computed tomography 21 months after the initial operation. She declined further investigations due to advanced age and was placed under close surveillance. Cases harboring a higher CD26+ level (defined by ≥median, i.e., 3.3%) appeared to correlate with the presence of confirmed or suspected metastases (*p* = 0.061, Table 2). The CD26+ proportion was 7.20 ± 5.20% in tumors with suspected or confirmed metastasis, and was 0.43 ± 0.15% in those without, showing a tendency to be higher in the former group (*p* = 0.13, data not shown). Univariate analyses were performed to investigate the prognostic implication of age, gender, metastases, tumor size, degree of differentiation, and CD26+ population percentage (Table S1). We also studied whether the emergence of increasing CD26+ subpopulation correlated with any clinical parameters. For tumor size, degree of differentiation, and disease stage at operation, no correlation with CD26+ level was found (*p* > 0.05, Pearson correlation). The site of metastases was also not found to be related to CD26+ level (Figure S1).

### 2.2. PIK3CA Inhibitor Decreases the Maintenance of CD26+ Cell Population in Culture

**Figure 2:**
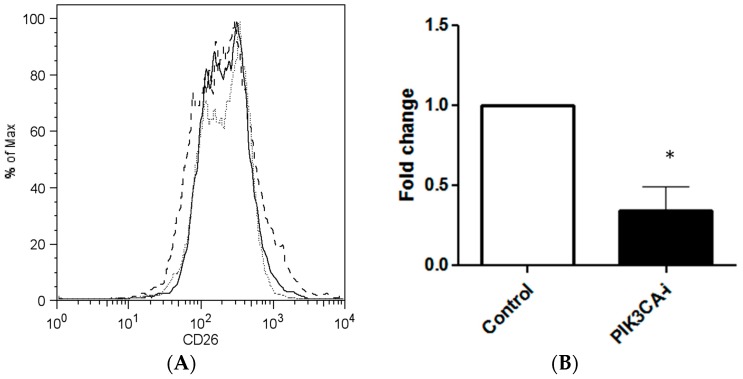
PIK3CA inhibitor decreases CD26+ stem cell population in colorectal cancer cell line. (**A**) Representative flow cytometry plots after treatment with PIK3CA-inhibitor in HCT116, showing decrease of CD26+ cell population. Grey dotted line, isotype control. Black dashed line, untreated. Black solid line, treated with PIK3CA-inhibitor. CD26 positivity was defined by >99th percentile of CD26 intensity stained with isotype control. (**B**) Bar chart shows decrease in CD26+ population after PI3KCA-inhibitor treatment in HCT116 cell line. Bar = Mean + SEM. *p* = 0.048, *n* = 3, * *p* < 0.05.

We hypothesized that CD26+ cells emerged from non-stem colon cancer cells under the influence of genes which characterize the late stage of colorectal carcinogenesis. We investigated whether an inhibition of *PIK3CA* may decrease the CD26+ subpopulation within colorectal cancer cells. PIK3CA inhibitor was added to cultures of cells lines HCT116 and SW480, and the proportion of CD26+ cells of the resultant culture was measured by flow cytometry after an incubation period of 3–5 days. In HCT116, the baseline proportion of CD26+ cells in HCT116 was 6.17%, which was reduced to 1.67% after the addition of PIK3CA inhibitor, translating to an overall decrease of CD26+ cells by 66.0% (*p* = 0.048, *n* = 3) (Figure 2). Experiments were also performed on additional cell lines. For SW480, because the baseline (control) proportion of CD26+ cells was small, this limited the possible decrease of CD26+ cells population by the modulation of PIK3CA inhibitors, and was too modest to be statistically significant (Figure S2A). For SW48, PIK3CA inhibitors decreased the CD26+ level to about 0.75 ± 0.29-fold (Figure S2B, *n* = 3, *p* = 0.28). As was the case for SW480, the low baseline level of CD26+ restricted the possible effect of PIK3CA inhibitors and thus may have impacted the statistical significance. 

### 2.3. TP53 Knockdown Increases the Maintenance of CD26+ Cell Population in Culture

**Figure 3:**
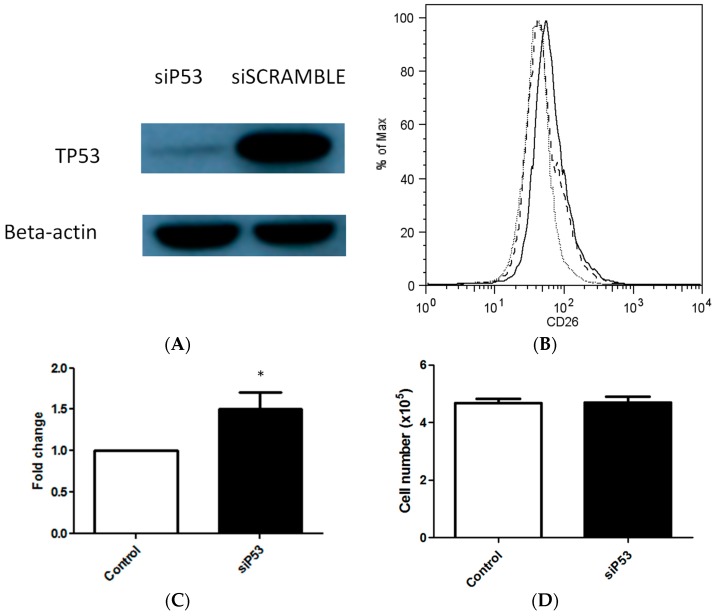
*TP53* knockdown in colorectal cancer cell results in a greater proportion of CD26+ cell population. (**A**) *TP53* knockdown with si*P53* as identified by Western blot. (**B**) *TP53* knockdown leads to a greater proportion of CD26+ cancer stem cells. Representative flow cytometry plots after treatment with si*P53* in SW480. Grey dotted line, isotype control. Black dashed line, Control transfected with siSCRAMBLE. Black solid line, treated si*P53*. CD26 positivity was defined by >99^th^ percentile of CD26 intensity stained with isotype control. (**C**) Bar chart shows increase in CD26+ population after si*P53* treatment in SW480 cell line. Bar = Mean + SEM. *p* = 0.047, *n* = 7. * *p* < 0.05. (**D**) *TP53* knockdown did not significantly alter cell number after 48 h in SW480 cell culture. *p* > 0.05, *n* = 6.

To investigate whether the tumor suppressor *TP53* could influence the percentage of CD26+ cells found in the resultant cultures, *TP53* was knocked down within the SW480 cell line using a targeting small interfering RNA (siRNA). The maximal knockdown as evidenced by Western blot was 48 h after transfection (Figure 3A). *TP53* knockdown increased the percentage of the resulting CD26+ cells by 1.50-fold, from the baseline of 1.83% to that of 2.51% after transfection, suggesting that either CD26+ cells production or maintenance was increased (*p* = 0.047, *n* = 7) (Figure 3B,C). To investigate whether the increase in the proportion of cells expressing the CD26+ phenotype may be due to an increased proliferation rate of the cells after *TP53* knockdown, the number of cells harvested at the end-point of the experiments was counted. *TP53* knockdown did not appear to have increased the rate of cell proliferation in SW480 (*p* > 0.05, *n* = 6) (Figure 3D).

Because the cancer stem cell population in a cancer is by definition small, the effect exerted on this intricate cell population by the knockdown of a tumor suppressor gene could be minute. When another cancer cell line, SW620, was tested, the change in CD26+ cell population was about 3.88 ± 3.42-fold (Figure S3). 

## 3. Discussion

The origin of cancer stem cells is still unsettled in the research community. Possibilities include tumor daughter cells, transit amplifying cells, or tissue stem cells [9,10]. The discovery of CSC with metastatic properties further complicates the picture [11]. Our study aims to provide some preliminary evidence on this topic. 

Our group previously demonstrated that a CD26+ tumor cell subpopulation in primary tissue has stem-like properties in that these cells can initiate sphere formation and tumor formation in serial dilution implantation experiments [2]. We have also shown that CD26+ correlates with the clinical stage on immunohistochemistry [4]. This study shows that confirmed or suspected stage IV primary tumors have a relatively large CD26+ subpopulation, while in stage I to III disease there is no apparent trend of the CD26+ population increasing with the increase of the disease stage. One argument is that the emergence of CD26+ may be an event that occurs as late as after stage III in the disease, just before metastasis sets in. There is a possibility that micrometastases may have occurred in a patient with an initial low pathological TNM stage disease. Also, the high percentage of CD26+ cancer cells in two patients later found to have confirmed or suspected metastases may imply the role of CD26+ on occult micrometastases. However, we are cautious while interpreting these preliminary observations. 

*TP53* is a tumor suppressor gene, whose mutation is known to be a late event and is implicated in around 70% of colorectal cancer cases [5]. *PIK3CA* is an oncogene found to be mutated in around 25% of tumors and is also a late event [7]. Our study showed that the knockdown of *TP53* enlarges the CD26+ population, while inhibition of *PIK3CA* has the opposite effect. We further hypothesized that this CD26+ cell population had stem-like properties, and could arise in a colorectal tumor with a predominant population of CD26− daughter cells at the late stage. 

To this end, we used fluorescent-activated cell sorting to select CD133−/CD26− cells for culture using colorectal cancer cell lines HCT116 and HT29. After a period of 7 to 14 days, sphere formation was detected in CD133−/CD26− cell cultures from both cell lines, indicating the formation of cells with stem cell-like properties (Figure S4C). Cells were later retrieved from the cultures (including spheres and supernatant) and subjected to flow cytometry, confirming the emergence of CD26+ cells (Figure S4A,B). Immunofluorescence staining was carried out (Figure S4D), and demonstrated the presence of both CD133+ and CD26+ cells in the tumor sphere. Taken together, the results suggest that the tumor spheres containing CD26+ cells arise in a predominant CD26− colorectal cancer cell culture. When CD133+/CD26− sorted cells were cultured, this population consisting of presumed stem cell properties (CD133+) did not appear to produce more CD26+ tumor cells than the CD133−/CD26− population (Data not shown). Unless a single cell sorting was carried out, cross-contamination between different populations remains a possibility. However, even if modest CD26+ cell contamination occurred in the CD26− cell culture, these CD26+ cells did not appear to be selectively enriched under the culture condition, as a significant proportion of CD133−/CD26− cells were still present after the culture period. 

Taken together, the emergence of the CD26 cell surface marker allows for two possible interpretations; either the cells has acquired stem-like properties during the cell culture, or the change in the cell surface marker is merely an effect of non-genetic phenotype plasticity in response to environmental stress [12]. However, by manipulating the expression of cancer genes implicated in the late stage of colorectal carcinogensis, *PIK3CA* and *TP53*, we observed that the CD26+ population with stem-like properties can be initiated or maintained. This finding does not favor the mechanism of non-genetic phenotype plasticity, although this possibility is not completely excluded. Also, the increase in CD26+ CSC subpopulations can be due to either an increase in cell formation, an increase in cell survival, or a decrease in cell differentiation. Each mechanism likely contributes to some extent to our observation. As CD26− and CD26+ cells may interchange in the cell culture due to some inherent genetic changes, each population should not be viewed in isolation but in a dynamic equilibrium. 

Various research groups have shown that differentiated cells may spontaneously convert to stem cells [13,14]. Provided that daughter cells greatly outnumber stem cell populations in a given tumor, the contribution of daughter cells to the generation of stem cells may be significant. This study suggests the dynamic relationship between daughter cancer cells and stem cells, and also the relationship between metastatic colon cancer stem cells and late genes in carcinogenesis. This has several implications. The evidence is in line with the established postulate that colon cancer occurs in a long sequence with the addition of genetic aberrations over many years [5,15,16,17], with metastatic properties acquired at late stage [5]. If the generation of CD26+ metastatic CSC occur at a late stage, early treatment of colon cancer may not leave latent micrometastatic foci in a patient.

We understand the limitations of this study include several aspects. While the CD26+ level is expected to be high in tumors with confirmed or suspected metastases, it is also important from the clinical point of view to quantify CD26+ levels in patients with early stages of cancer in the hope of identifying those who may be more prone to developing metastatic disease. It remains unknown whether a high CD26+ level in early-stage tumors represents the possibility of undetected micrometastases or truly increased risks of future metastasis, or whether this is merely an incidental finding. The limitation in obtaining viable cell samples from clinical specimens for flow cytometry, which is particularly true for the inherently contaminated colorectum, complicated the analyses. Future investigations correlating the CD26+ level of the primary tumor with quantities of circulating tumor cells or cell-free nucleic acid may be useful. 

Limitations were also identified for the in vitro studies. First, cancer cell lines may have acquired many more genetic aberrations with passages compared to cancer cells in vivo. Second, the percentage of CD133+ and CD26+ cells were found to be much greater in cancer cell lines than in tissue obtained from patients. Third, the in vitro model is at its best a simulation of its in vivo counterpart, where the tumor microenvironment may contribute to malignant cell growth, maintenance, and metastasis [18,19]. We discussed above that contamination during cell sorting may not be a significant confounder to the current analysis. Nevertheless, in order to definitively examine the notion that cancer stem cells can arise from daughter tumor cells, the single cell sorting of CD26− cells may eventually be required, and the number of single cells required for this purpose is expected to be very large. Meanwhile, if the implantation of pure tumor (non-stem) cells in mice can give rise to stem cells, the cancer bulk can in theory be maintained. Further cell sorting for stem cells and limiting dilution assays would be definitive. These are important research directions. As for the current preliminary observations, we believe the in vitro finding that CD26+ CSC can emerge from CD26− daughter cells suggests this conversion may well occur in patients.

The theory of cancer stem cells has casted doubt on the current treatment for cancer, because chemotherapy at its best only debulks the tumor, while many of the CSC, critical to tumor maintenance but more resistant to chemotherapy [20,21,22], are left untouched. Our study supports the current rationale of surgical debulking and adjuvant chemotherapy of colon cancer. If a vast majority of tumor daughter cells are removed, the niche from which CSC can form decreases vastly. We believe that gaining knowledge on the interaction between cancer stem cells and daughter cells will continue to shift the paradigm in stem cell research and patient care.

## 4. Materials and Methods

### 4.1. Cell Culture

HT29, SW480, SW48, SW620 and HCT116 (obtained from American Type Culture Collection (ATCC), Manassas, VA, USA) were maintained in Dulbecco’s Modified Eagle’s Medium (DMEM) (Life Technologies, Carlsbad, CA, USA) and supplemented with 10% fetal bovine serum (FBS) (Life Technologies) and 1% Penicillin-Streptomycin (Life Technologies), and placed in an incubator with a temperature of 37 °C, under 5% CO_2_ humidity. For sphere formation, cancer cell obtained from fluorescent-activated cell sorting (FACS) were cultured on ultra-low attachment plates (Corning Inc., Corning, NY, USA), and also incubated under the aforementioned conditions.

### 4.2. Flow Cytometry and Fluorescent-Activated Cell Sorting (FACS)

Cells were harvested from culture, washed with phosphate buffered saline (PBS), and incubated with the appropriate staining antibodies in binding buffer for 15 min at room temperature in the dark. After being washed with PBS twice, the cells were analyzed on a Cytomics FC500 (Beckman Coulter, Fullerton, CA, USA) or BD FACSCalibur (BD Biosciences, San Jose, CA, USA)). Cell sorting was performed with a MoFlo XDP Cell Sorter (Beckman Coulter). In all cytometry analyses and cell sorting experiments, an appropriate isotype control was used, and the positive population was defined where the fluorescent signal intensity exceeded that of the 99th percentile of the isotype control. The data acquired were analyzed using FlowJo (version 8.7, Tree Star, Inc., Ashland, OR, USA). Antibodies used include CD133-PE (Miltenyi Biotech, Bergisch, Gladbach, Germany) and CD26−PE/Cy5 (Miltenyi Biotech). 

### 4.3. PIK3CA Inhibitor Assay

One hundred and fifty thousand cells per well were seeded in an adherent six-well culture plate (Corning Inc.) under the culture conditions described above. On the subsequent day, fresh antibiotics-free DMEM with 10 µM *PIK3CA* inhibitor (LY294002, Cell Signaling Technology Inc., Danvers, MA, USA) was used as a culture medium. Cells were harvested after 48 h for downstream analyses.

### 4.4. Knockdown of P53

Small interfering RNA (siRNA) targeting *p53* (s605, ThermoFisher Scientific, Waltham, MA, USA) at 40 nM was delivered to the cells using Lipofectamine 2000 (Invitrogen Inc., Carlsbad, CA, USA), according to the manufacturer’s instruction. Nonsense scrambled oligo (Stealth RNAi siRNA Negative Control, Invitrogen) was used as a control. Cells were harvested after 48 h post-transfection for analyses. For Western blot analyses, ice-cold radioimmunoprecipitation assay buffer (RIPA) buffer (Cell Signaling Technology, Danvers, MA, USA) containing phenoylmethylsulfonyl fluoride (1 mmol/L) and protease inhibitor was used for cell lysis. The lysate was centrifuged at 12,000× *g* for 15 min to obtain the supernatant. The harvested protein was suspended in sodium dodecyl sulfate buffer and resolved by sodium dodecyl sulfate—polyacrylamide gel electrophoresis. Transfer to polyvinylidene difluoride (PVDF) membranes (GE Healthcare, Piscataway, NJ, USA) was carried out. The antibody against TP53 was purchased from Cell Signaling Technology (Danvers, MA, USA). The membranes were developed after probing with horseradish peroxidase-conjugated secondary antibodies. 

### 4.5. Clinical Specimens

Fresh tumor resection specimens were obtained with informed consent from 11 colorectal cancer patients who underwent surgical resection at the Department of Surgery, Queen Mary Hospital, University of Hong Kong, approved by the Institutional Review Board of the University of Hong Kong/Hospital Authority Hong Kong West Cluster (HKU/HA HKW IRB). The specimens were immediately minced on ice, suspended in DMEM/F12 medium (Invitrogen, Carlsbad, CA, USA), and dissociated with collagenase (Invitrogen) and hyaluronidase (Calbiochem, La Jolla, CA, USA). Enzymatically disaggregated suspensions were filtered and washed three times with PBS. Red blood cells were removed by Histopaque-1077 (Sigma, St. Louis, MO, USA). The resulting single tumor cells were used for flow cytometry analyses.

### 4.6. Statistical Analysis

Continuous variables were expressed as means ± SD. Treatment groups were compared with the independent or paired sample t test where appropriate. Comparisons involving variables with non-normal distribution were performed by the Mann-Whitney U test. Comparisons of nominal variables were performed via Chi-square test or Fisher’s exact test where appropriate. *p*-Values < 0.05 were considered statistically significant. Analyses were performed with the SPSS 20 statistics software (version 20, SPSS Inc., Chicago, IL, USA).

## 5. Conclusions

Knowledge of the timing and cell type where cancer stem cells with metastatic properties may arise is critical for effective treatment, although this probably varies with different cancers. In colorectal cancer, disease develops over a long period of time by stepwise acquisitions of mutations which are relatively well-defined. Detectable metastases usually occur as the terminal event. This study supports the possibility that emergence of CD26+ colorectal cancer stem cells with metastatic properties can be under the influence of genes that are usually mutated late in the disease course.

## Figures and Tables

**Table 1:** Flow cytometry analysis of CD26+ cancer cells in colorectal cancer patients and clinical data.

Patient	Gender and Age	CD26+ Cells %	Stage at Surgery	Tumour Size (cm)	Degree of Differentiation	Metastasis Diagnosed or Suspected	Survival Status	Metastasis-Free Survival (Months)	Overall Survival (Months)
1	M/81	0.2	III	9	Moderate	Liver	Deceased	5.40	9.70
2	F/80	0.3	I	5	Moderate	None	Alive	39.23	39.23
3	F/68	0.4	IIA	10	Moderate	None	Alive	36.20	36.20
4	M/71	0.5	IIIC	5	Moderate	Peritoneum and bone	Deceased	13.97	19.83
5	M/79	0.6	IIA	11	Poor	None	Alive	33.17	33.17
6	M/83	3.3	IV	12	Moderate	Lung	Deceased	NA	19.47
7	M/74	8.2	IV	5	Poor	Liver	Deceased	NA	51.97
8	F/56	9.6	IV	5.5	Moderate	Liver	Deceased	NA	29.67
9	M/68	9.9	IV	9	Moderate	Lung and liver	Alive	NA	47.50
10	F/84	12.7	IIA	10	Moderate	Suspected lung metastasis	Alive	21.30	31.23
11	M/78	13.2	IIA	15	Moderate	Liver	Deceased	5.10	5.17

CD26+ positive cells were defined by >99th percentile of CD26 intensity stained with isotype control. For the tumor size, the largest tumor dimension was given. All patients underwent surgery for colorectal cancer, whereas for patients 6, 7, 8, and 9, surgery for both the primary and metastatic tumors were performed. For patients 1, 4, 10, and 11, metastatic disease was suspected or confirmed after the initial surgery. NA, not applicable.

**Table 2:** Presence of confirmed or suspected metastases correlated with higher CD26+ cell proportion.

Number of Patients		Confirmed or Suspected Metastases	
		Absent	Present
CD26+ cell proportion	low	3	2
	high	0	6

Presence of confirmed or suspected metastases correlated with higher CD26+ cell proportion (≥ median, which is 3.3% of CD26+ level). *p* = 0.061, Fisher exact test.

## Supplementary Materials

Supplementary materials can be found at www.mdpi.com/1422-0067/18/6/1106/s1.
