# Supplementary material for: Emergence of CD26+ Cancer Stem Cells with Metastatic Properties in Colorectal Carcinogenesis
Source: Int J Mol Sci. 2017 May 23;18(6):1106. doi: 10.3390/ijms18061106 (PMC5485930; doi:10.3390/ijms18061106)
Supplement: Supplementary file 1 [file ijms-18-01106-s001.pdf]

Variables	Metastasis-free survival		Overall survival	
	Hazard ratio	P-value	Hazard ratio	P-value
CD26+ level	1.051	0.588	1.005	0.957
Tumor size	1.091	0.487	1.186	0.33
Female gender	0.64	0.592	0.291	0.274
Age	1.028	0.509	1.003	0.958
Poor tumor differentiation	0.473	0.484	0.454	0.033
Presence of metastasis	N/A	N/A	40.41	0.35

SUPPLEMENTARY TABLE 1

Univariate analyses showing hazard ratio of various clinical parameters on reduced metastasis-free survival and overall survival. N/A, not applicable

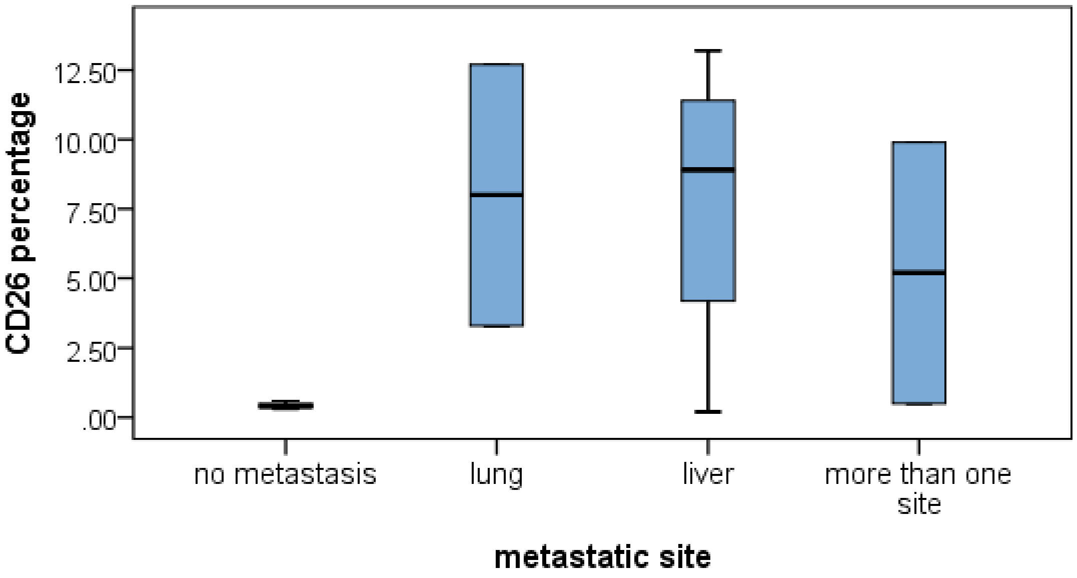

SUPPLEMENTARY FIGURE 1

CD26+ tumor cell count according to metastatic sites. No statistical difference was found among different metastatic sites.

A B

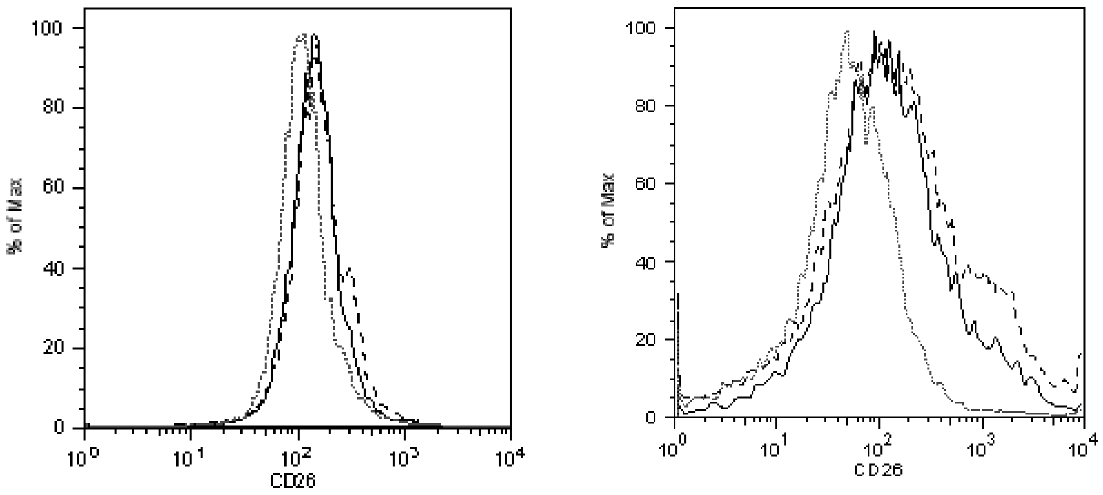

SUPPLEMENTARY FIGURE 2

Representative flow cytometry plots of colorectal cell lines (A) SW480 and (B) SW48 treated with PI3KCA-inhibitor. Grey dotted line, isotype control. Black dashed line, untreated. Black solid line, treated with PIK3CA-inhibitor.

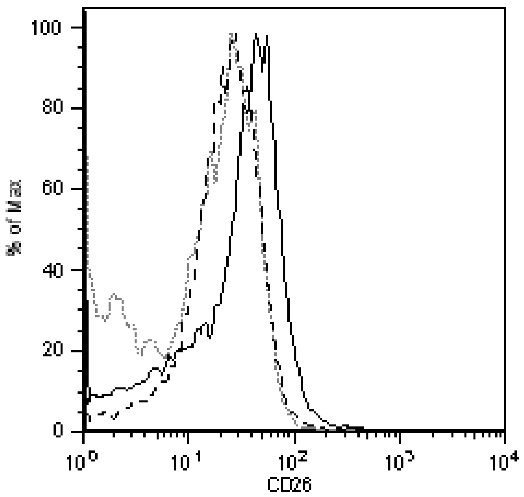

SUPPLEMENTARY FIGURE 3

Representative flow cytometry plot of colorectal cell line SW620 treated with siTP53. Grey dotted line, isotype control. Black dashed line, untreated. Black solid line, treated with siTP53.

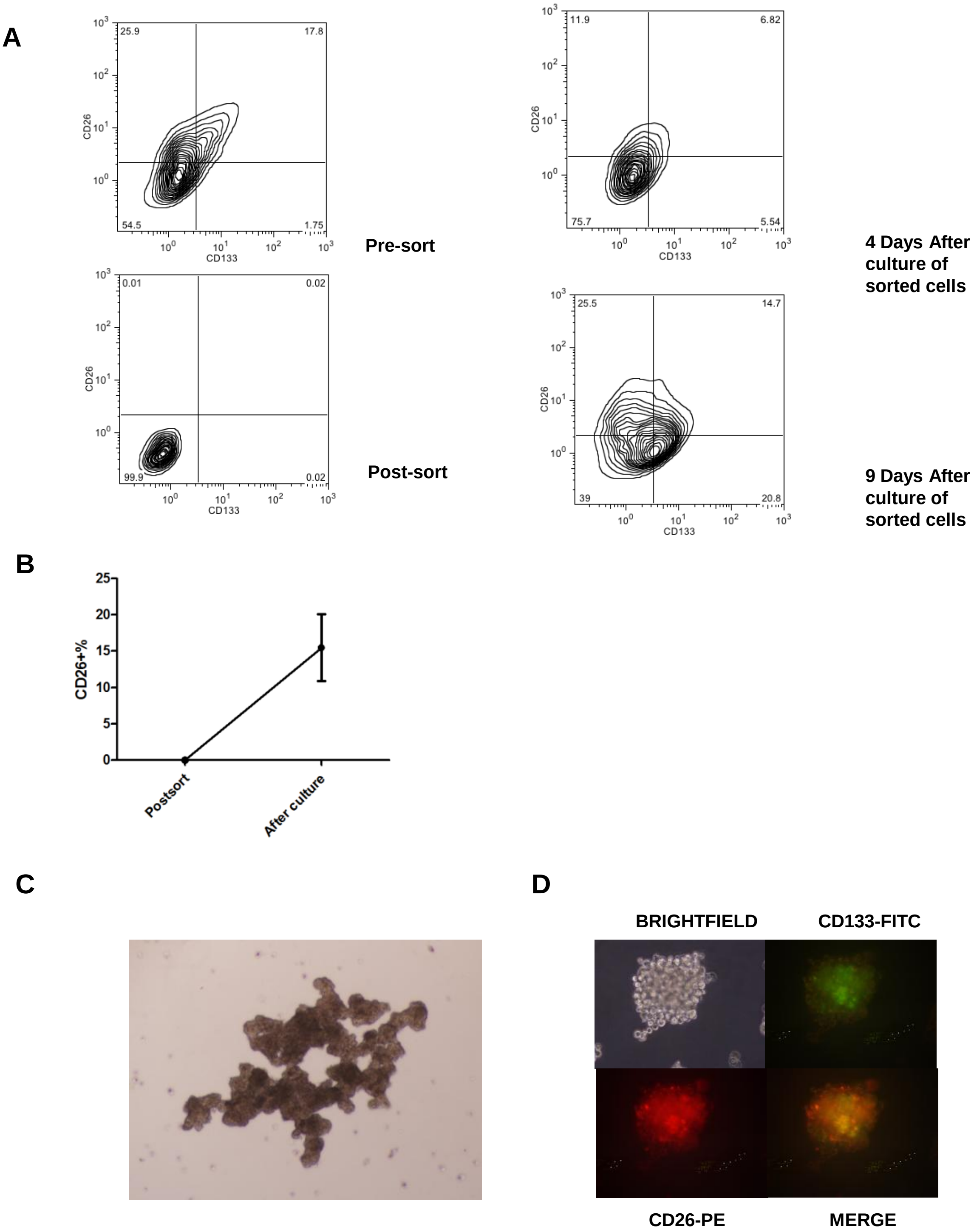

Supplementary figure 4 CD26+ cancer stem cells may emerge from non-cancer stem cells.

(A) Sorting CD133-CD26- cell population and suspension culture. Gating was designed to select at most 10% of cells which best represented the CD133-CD26- population. Post-sort flow cytometry indicates that predominant CD133-CD26- were sorted. Flow cytometry carried on suspension culture of sorted cells on day 4 and 9 after cell sorting indicates emergence of CD133+ and CD26+ population. Numbers at the corners of four quadrants indicate percentage of cells in the respective quadrant. Representative flow cytometry figure of cell line HT29.

(B) Change of CD26+ population after culture of CD133-CD26- sorted cells for 4 days.

(C) Representative spheres in sphere formation assay of the sorted CD133-CD26- cells.

(D) Presence of CD133+ and CD26+ cells in tumour spheres. Immunofluorescence staining was carried out with CD133-FITC and CD26-PE, showing presence of positive cells in tumour spheres.
